# Draft Genome Sequence of *Mycobacterium heraklionense* Strain Davo

**DOI:** 10.1128/genomeA.00807-15

**Published:** 2015-07-23

**Authors:** Alexander L. Greninger, Gail Cunningham, Charles Y. Chiu, Steve Miller

**Affiliations:** Department of Laboratory Medicine, UCSF, San Francisco, California, USA

## Abstract

We report the draft genome sequence of *Mycobacterium heraklionense* strain Davo, isolated from a fine-needle aspirate of a right-ankle soft-tissue mass. This is the first draft genome sequence of *Mycobacterium heraklionense*, a nonpigmented rapidly growing mycobacterium.

## GENOME ANNOUNCEMENT

*Mycobacterium heraklionense* is an unpigmented member of the *Mycobacterium terrae* complex that was originally isolated from Heraklion, a city in Crete, Greece (1). It is an uncommon cause of infection of humans, with the first case reported only in 2014 (2). *Mycobacterium heraklionense* has also been reported to have been isolated from water treatment sludge (3). The notable phenotypic characteristics of *Mycobacterium heraklionense* include an intermediate growth rate, smooth colonies with no pigmentation, positive nitrate reduction, and positive beta-glucosidase activity (1).

Rapidly growing mycobacteria constitute a commonly isolated population of acid-fast bacilli in the clinical microbiology lab of varying clinical importance, and molecular methods are often the only means of rapidly discriminating between them (4, 5). We sequenced the first draft genome of *Mycobacterium heraklionense* from a fine-needle aspiration sample from a 53-year-old woman, who presented to an orthopedic surgery clinic in 2014 with a right-medial soft-tissue ankle mass. The patient had previously had a repair of a transverse laceration of her right Achilles tendon after a gardening accident 20 years prior. Cytological analysis of the aspirate revealed purulent fluid consistent with an inflamed cyst, and the acid-fast bacillus culture returned positive on day 14 of culture. The isolate was originally typed as *Mycobacterium heraklionense* based on partial *rpoB* sequencing. Susceptibility testing showed the following MICs (μg/mL): amikacin, ≤2; ciprofloxacin, >4; clarithromycin, ≥4; ethambutol, 1.25; kanamycin, 8; moxifloxacin, >2; rifabutin, 0.25; rifampin, >16; and streptomycin, 4. No other bacteria or yeast were recovered from the aspirate.

DNA from *M. heraklionense* strain Davo was extracted using the Qiagen EZ1 kit, and paired-end libraries were prepared using the Nextera XT DNA library kit, followed by 2 × 250-bp sequencing on Illumina HiSeq. Sequences were adapter and quality trimmed (Q30) using Cutadapt, *de novo* assembled using SPAdes version 3.5, metagenomically screened for contaminating sequence with SURPI, and annotated via Prokka version 1.1 (6–9). A total of 11,595,000 paired-end reads with an average length of 181 nucleotides were recovered after trimming. *De novo* assembly yielded 75 contigs for a total assembly size of 5,109,749 bp with an *N*_50_ of 187,081 bp, an average coverage of 337×, and a total of 4,742 coding sequences. Contiguity was most likely disrupted by the high GC content (68%), along with several high-copy-number integrases, transposases, and recombinases that were longer than the sequence read length. A 55.5-kb contig that was present at a 20-fold-higher copy number than other genomic contigs demonstrated 65% nucleotide identity to a variety of *Mycobacterium* phages, including Saal, Sparky, and CaptainTrips (10).

BLASTn analysis of the complete 16S sequence from strain Davo showed 100% identity to *M. heraklionense* type strain NCTC 13432, while other top hits included members of the *M. terrae* complex, including *M. arupense*, *M. senuense*, and *Mycobacterium* sp. JDM601. No high-confidence antibiotic resistance genes were identified by Comprehensive Antibiotic Resistance Database analysis (11).

### Nucleotide sequence accession numbers.

This whole-genome shotgun project has been deposited at DDBJ/ENA/GenBank under the accession number LDPO00000000. The assembly described in this paper is the first version, LDPO01000000.
